# Does Acupuncture Produce Durable Analgesia in Trigeminal Neuralgia? An Updated Systematic Review and Meta-Analysis

**DOI:** 10.3390/healthcare14131926

**Published:** 2026-07-01

**Authors:** Wei Wang, Weiming Wang, He Chen, Xinyu Shen, Jiarong Fan, Shuai Gao, Zhishun Liu

**Affiliations:** 1 Graduate College, Beijing University of Chinese Medicine, Beijing 100029, China; kenzie121320@outlook.com (W.W.);; 2 Department of Acupuncture, Guang’anmen Hospital, China Academy of Chinese Medical Sciences, Beijing 100053, China

**Keywords:** trigeminal neuralgia, acupuncture, meta-analysis, risk of bias, durable effect, recall period

## Abstract

**Highlights:**

**What are the main findings?**

- End-of-treatment pain intensity estimates favoring acupuncture were statistically significant only in high risk-of-bias trials and were compromised by pervasive recall period omission. Follow-up data showed a directionally consistent but statistically fragile signal of analgesic persistence at three months; the pooled estimate was not robust to single-study removal and is hypothesis-generating only.

**What are the implications of the main findings?**

- The favorable safety profile relative to carbamazepine is the most robust finding, but current evidence does not support routine clinical adoption, and the durable-effect signal remains exploratory pending confirmatory trials. Future trials require sham controls, prospective pain diaries, and follow-up as a co-primary endpoint to determine whether this signal reflects a true durable effect.

**Abstract:**

**Background/Objectives:** Trigeminal neuralgia (TN) is a relapsing-remitting condition predominantly managed in primary care, where carbamazepine remains the sole recommended first-line therapy. Prior meta-analyses report end-of-treatment pain reductions with acupuncture but have not stratified results by risk of bias, audited pain outcome reporting quality, or examined whether analgesic effects persist beyond the treatment period. This updated systematic review addressed these gaps. **Methods:** Seven databases were searched from inception to February 2026 for randomized controlled trials comparing acupuncture against non-acupuncture controls in adults with TN (PROSPERO: CRD420251271469). End-of-treatment VAS, follow-up VAS, attack frequency, recurrence rate, and adverse events were analyzed using random-effects models with Hartung–Knapp–Sidik–Jonkman adjustment. Risk of bias was assessed with Cochrane RoB 2. Recall period specification was audited for all pain outcomes. **Results:** Twenty-three trials (1774 participants) were included, all from China, with one sham-controlled comparator. Fewer than 15% of VAS outcomes specified a recall period. End-of-treatment pain intensity favored acupuncture (MD = −1.49; 95% CI −1.81 to −1.16; I^2^ = 93.1%), but the prediction interval crossed zero and significance was confined to high-risk studies (k = 16; *p* < 0.001). At three-month follow-up, the pooled estimate was comparable in magnitude (k = 3; MD = −1.50; I^2^ = 47.0%) but was not robust to single-study removal. Recurrence rate favored acupuncture in both reporting studies. Acupuncture was associated with fewer adverse events than carbamazepine (RR = 0.31; I^2^ = 0%). **Conclusions:** End-of-treatment estimates are compromised by risk-of-bias dependence and pervasive recall period omission. Follow-up data showed a directionally consistent but statistically fragile signal of analgesic persistence. Sham-controlled trials with prospective pain diaries and follow-up as a co-primary endpoint are needed.

## 1. Introduction

Trigeminal neuralgia (TN) presents as paroxysmal, electric-shock-like unilateral facial pain triggered by innocuous stimuli, affecting 0.03% to 0.3% of the population and predominantly those older than 50 years [1,2,3]. The condition imposes substantial psychological burden, with comorbid anxiety, depression, and impaired daily activities well documented [4,5]. Critically, approximately two-thirds of patients follow a relapsing-remitting course, with spontaneous remissions lasting months to years [6,7]. This episodic trajectory has direct implications for how treatment outcomes should be defined and measured.

Current guidelines recommend carbamazepine (CBZ) as first-line therapy, with oxcarbazepine as an alternative [8]. However, fewer than half of patients achieve sustained relief. Adverse effects including cognitive impairment, dizziness, and hyponatremia are frequent, particularly in the elderly population where TN incidence peaks [9,10]. When first-line therapy fails, second-line oral options including gabapentin, pregabalin, baclofen, and lacosamide are frequently used [11]. Surgical alternatives carry risks of sensory loss and recurrence [12]. For patients managed in primary care, where most initial prescribing in this setting occurs, treatment options are, therefore, largely confined to CBZ and its attendant toxicities [13]. Acupuncture, with its established safety profile in chronic pain management, has attracted growing interest as an adjunctive approach, and several systematic reviews have evaluated its analgesic efficacy.

Existing meta-analyses consistently report that acupuncture reduces pain intensity relative to CBZ at end of treatment, with fewer adverse effects [14,15,16]. Yet all prior syntheses evaluated only end-of-treatment pain scores. None examined whether analgesic benefit persisted beyond the active treatment period. For a relapsing-remitting condition, this is the more clinically relevant question. In addition, substantial unexplained heterogeneity was present in every prior pooled estimate. A recent overview further identified that no existing synthesis adequately assessed the impact of risk of bias on pooled results [17].

This gap in outcome selection is at odds with patient priorities. The TN Core Outcome Set (TRINCOS) Delphi consensus identified “duration of pain relief” as the single outcome rated critical by all participating patients, above point-in-time pain intensity [18]. No prior review has examined acupuncture’s effects on follow-up pain, attack frequency, or recurrence. A parallel measurement concern reinforces this gap. VAS pain intensity is sensitive to the recall period specified to patients [19]. In TN, where spontaneous remission is common, a score obtained without a defined recall window may capture disease fluctuation rather than treatment response [6,7]. The European Medicines Agency (EMA) guideline on analgesic development requires that pain assessment accommodate the temporal course of the condition under study, including paroxysmal presentations [20]. Most published acupuncture trials for TN did not meet this requirement.

We, therefore, conducted an updated systematic review and meta-analysis. Our search extended to February 2026, incorporating five trials published after the most recent prior search date, including the first sham-controlled trial in this literature [21]. Beyond end-of-treatment pain intensity, we examined follow-up pain, attack frequency, and recurrence rate. We also audited pain outcome reporting quality, stratified pooled estimates by risk of bias, and explored sources of heterogeneity through subgroup analyses and meta-regression.

## 2. Material and Methods

This study followed the Preferred Reporting Items for Systematic Reviews and Meta-Analyses (PRISMA) extension guidelines for meta-analysis and the Cochrane Handbook for Systematic Reviews of Interventions. It was prospectively registered in PROSPERO (CRD420251271469).

### 2.1. Data Sources and Searching

Two investigators independently conducted a comprehensive systematic search across seven electronic databases: PubMed, Embase, Web of Science, Chinese Biomedical Literature Database (CBM), China National Knowledge Infrastructure (CNKI), Wan-Fang Database, and Chinese Scientific Journal Database (VIP). The search period spanned from the inception of each database to 20 February 2026.

The search strategy employed a combination of MeSH terms and free-text words. The core search terms included: (1) Disease: “Trigeminal Neuralgia” OR “Tic Douloureux” OR “Trigeminal Pain”; (2) Intervention: “Acupuncture” OR “Electroacupuncture” OR “Needling” OR “Acupoint”; and (3) Study Type: (“Randomized Controlled Trial” OR “Clinical Trial” OR “RCT”). The full search strings for each database are provided in Table S1.

The search was limited to studies published in English or Chinese. Furthermore, the reference lists of the retrieved reviews and original studies were manually screened for any additional relevant publications. Any disagreements regarding study inclusion were resolved through consensus or by consulting a third senior investigator.

### 2.2. Eligibility Criteria

Studies were included according to the following PICOS criteria [22]. Adults diagnosed with trigeminal neuralgia according to recognized clinical diagnostic criteria were eligible. Studies involving patients with secondary trigeminal neuralgia caused by structural lesions (e.g., tumors or multiple sclerosis) were excluded. The intervention of interest was acupuncture-based therapy, including manual acupuncture (MA) or electroacupuncture (EA), administered either as monotherapy or in combination with conventional pharmacological treatment. Studies investigating other acupuncture-related techniques (e.g., acupressure, laser acupuncture, or transcutaneous electrical stimulation without needle insertion) were excluded. Eligible control interventions included conventional pharmacological treatments (e.g., carbamazepine or other medications), sham acupuncture, placebo interventions, or usual care. Sham acupuncture refers to non-penetrating or non-acupoint needling used to control for non-specific effects. Trials comparing different acupuncture techniques without a non-acupuncture control group were excluded. Studies were required to report at least one pain-related outcome. The primary outcome was pain intensity measured by validated scales such as the VAS or SF-MPQ. Secondary outcomes included pain intensity at follow-up (assessed at least one month after the end of active treatment), weekly attack frequency, duration of individual pain attacks, recurrence rate, and adverse events. Follow-up outcomes were classified by time point as reported in each trial. The inclusion and exclusion criteria are summarized in Table S2.

### 2.3. Data Extraction

Data were independently extracted from all eligible studies by two reviewers using a predefined form. Extracted items included participant demographics (sample size, mean age, and sex), intervention parameters (acupuncture type, acupoint selection, and treatment schedules), comparator characteristics (pharmacological type, dosage, and modality), and outcome data at baseline, end of treatment, and each reported follow-up time point. For pain intensity outcomes, the instrument name, scale range, anchor descriptors, and recall period specification were additionally recorded. For attack frequency, values were extracted as weekly counts; when reported as total counts over an observation window, values were converted to a weekly rate by dividing by the number of weeks. For recurrence outcomes, event counts and group denominators were extracted at each reported time point. Classification of outcome constructs was conducted independently by two reviewers, with discrepancies resolved through discussion.

### 2.4. Risk of Bias in Included Studies

Risk of bias in the included clinical trials was assessed using the Cochrane RoB 2 tool, covering five domains: the randomization process (D1), deviations from intended interventions (D2), missing outcome data (D3), measurement of the outcome (D4), and selection of the reported result (D5) [23]. Two reviewers conducted the assessments independently, and disagreements were resolved by consensus or consultation with the corresponding author.

### 2.5. Statistical Analysis

All statistical analyses were conducted using R (version 4.5.0) with the meta and metafor packages [24,25]. Continuous outcomes were summarized as mean differences (MDs) with 95% confidence intervals (CIs), or standardized mean differences (SMDs) when different scales were used [26]. For dichotomous outcomes, risk ratios (RRs) with 95% CIs were calculated. Change-from-baseline values were preferentially extracted. When unavailable, change scores were derived from baseline and post-intervention data, assuming a correlation coefficient of 0.5 in accordance with Cochrane recommendations [27,28]. To assess the influence of this assumption, sensitivity analyses were performed using alternative correlations of r = 0.3 and r = 0.7. Random-effects models were applied throughout, with between-study variance (τ^2^) was estimated using restricted maximum likelihood (REML). Confidence intervals were derived using the Hartung–Knapp–Sidik–Jonkman adjustment [29,30]. Statistical heterogeneity was assessed using the I^2^ statistic and Cochran’s Q test. I^2^ values of 0% to 40% were considered potentially unimportant, 30% to 60% moderate, 50% to 90% substantial, and 75% to 100% considerable [31]. In addition, 95% prediction intervals (PIs) were calculated to estimate the expected range of treatment effects in future studies when three or more studies were available [32]. Follow-up pain intensity outcomes were analyzed separately by time point. Data from different follow-up intervals were not pooled. When a single study reported data at multiple follow-up time points, only one time point was entered into each model to preserve statistical independence.

Sensitivity analyses were conducted using a leave-one-out approach. Pre-specified subgroup analyses were performed according to acupuncture type, overall risk of bias level, and control group drug dosage. Exploratory meta-regression examined session duration, weekly treatment frequency, and De qi requirement as candidate moderators. Publication bias was assessed by Egger’s regression test and funnel plot inspection when ten or more studies were available [33]. Certainty of evidence for each outcome was assessed using the GRADE framework, rating down from high (randomized trials) across five domains: risk of bias, inconsistency, indirectness, imprecision, and publication bias [34]. A two-sided *p* value < 0.05 was considered statistically significant.

## 3. Results

### 3.1. Search Results

**Figure 1:**
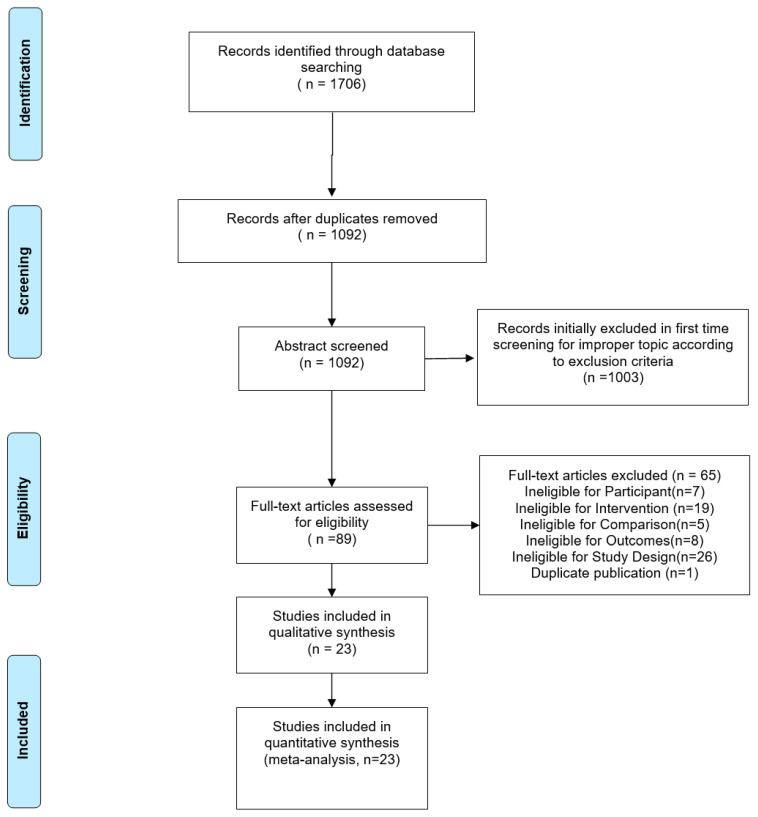
Literature screening process and results.

The literature search initially identified 1706 records. After removal of duplicates, 1092 records remained for title and abstract screening. Of these, 89 articles were assessed for full-text eligibility. A total of 23 randomized controlled trials met the inclusion criteria and were included in the qualitative and quantitative syntheses. The selection process is summarized in Figure 1.

### 3.2. Characteristics of Included Studies

The 23 included trials enrolled 1774 participants. Sample sizes ranged from 40 to 120. All studies were conducted in China and published between 2010 and 2024. Most trials compared acupuncture combined with CBZ or acupuncture alone against CBZ monotherapy; one trial used sham acupuncture combined with CBZ as the control [21]. Both MA and EA were investigated. Treatment duration was typically 4 weeks (range: 2 to 12 weeks), with most trials administering 7 sessions per week. Detailed study characteristics are summarized in Table 1 and Table S3.

Pain intensity was the primary outcome in all trials, assessed using VAS in 20 studies and SF-MPQ (including SF-MPQ-2) in 6 studies. Reporting quality of these instruments was poor. Among VAS outcomes, only 3 (15.0%) specified a recall period, all defining a 24-h window; scale range was unreported in 6 (30.0%) and anchor wording in 7 (35.0%). Among SF-MPQ outcomes, only Li 2024 [21] specified a recall period (two weeks); subscale reporting varied widely across the remaining five studies. Full reporting characteristics are presented in Tables S4–S6.

### 3.3. Risk of Bias Assessment

**Figure 2:**
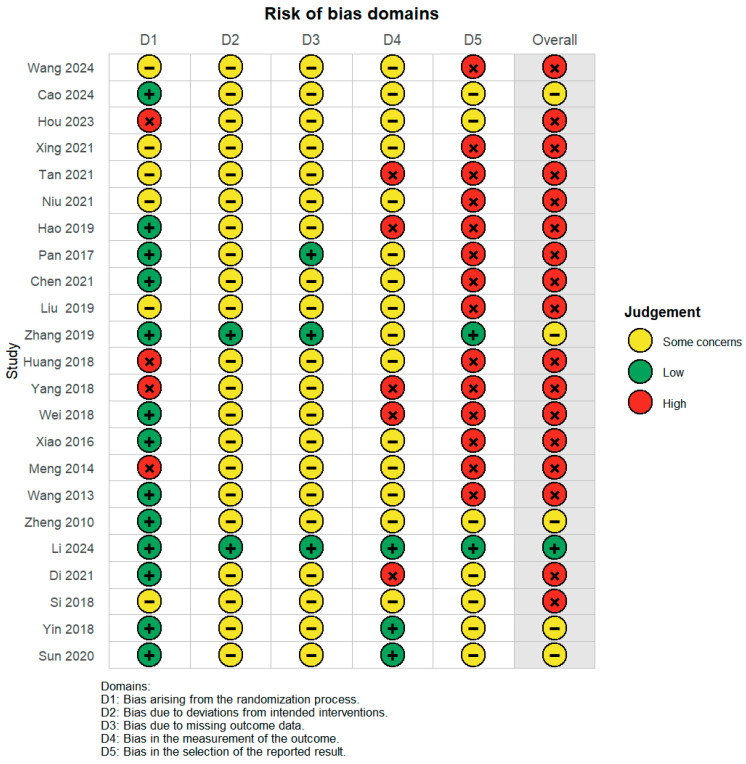
Risk of bias assessment of randomized controlled trials using the Cochrane RoB 2 tool [21,35,36,37,38,39,40,41,42,43,44,45,46,47,48,49,50,51,52,53,54,55,56].

**Figure 3:**
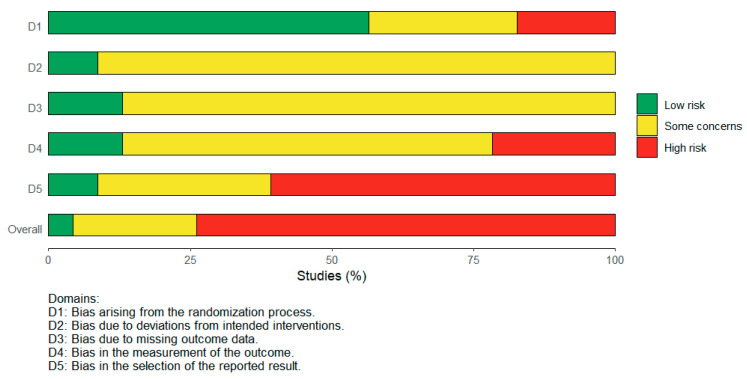
Summary of risk-of-bias judgments across domains for randomized controlled trials.

Of the 23 trials, one was rated low overall risk [21], five with some concerns [36,45,52,54,55,56], and the remaining 17 were rated high risk. The most common sources of high risk were D1 (randomization process), where four trials lacked adequate sequence generation or allocation concealment, and D4 (measurement of the outcome), where high risk ratings were concentrated in trials without a specified recall period for pain assessment. D5 (selection of the reported result) was frequently elevated owing to incomplete trial registration or discrepancies between registered and reported outcomes. D2 (deviations from intended interventions) and D3 (missing outcome data) were rated some concerns in most trials but contributed less to overall risk judgments. Full domain-level ratings are presented in Figure 2 and Figure 3.

### 3.4. End-of-Treatment Pain Intensity

#### 3.4.1. VAS Pain Intensity

- Meta-analysis of VAS pain intensity

Pooling 20 studies, acupuncture was associated with a significantly greater reduction in VAS pain intensity than controls (MD = −1.49; 95% CI −1.81 to −1.16; *p* < 0.001; I^2^ = 93.1%). The 95% prediction interval ranged from −3.09 to 0.02. Leave-one-out sensitivity analyses confirmed stability of the pooled estimate across all iterations (Figure S1). Egger’s regression test did not indicate significant publication bias (*p* = 0.23; Figure S2). Results were robust to the assumed correlation for imputing change scores. Pooled estimates were consistent across all three values (r = 0.3, MD = −1.40; r = 0.7, MD = −1.42; all *p* < 0.001; Table S7). A sensitivity analysis excluding the single sham-controlled trial [21] yielded a nearly identical pooled estimate from the 19 open-label trials (MD = −1.49; 95% CI −1.84 to −1.14; *p* < 0.001; I^2^ = 93.3%; Figure S3).

- Subgroup Meta-Analysis of VAS Pain Intensity

Subgroup analyses were conducted using random-effects models with the Hartung–Knapp–Sidik–Jonkman adjustment. Full results are presented in Table 2.

**Figure 4:**
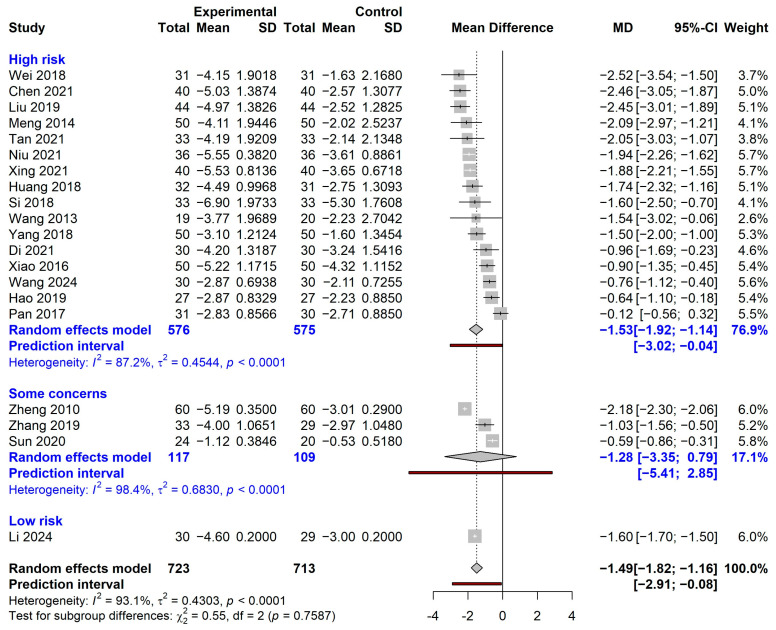
Forest plot of VAS pain intensity stratified by risk of bias [21,35,38,39,40,41,42,43,44,45,46,47,48,49,50,51,52,53,54,56]. Blue text denotes subgroup labels and pooled estimates; the red horizontal line indicates the prediction interval.

Overall risk of bias. The pooled effect was statistically significant in the high-risk subgroup (k = 16; MD = −1.53; *p* < 0.001) but not in the some-concerns subgroup (k = 3; MD = −1.28; *p* = 0.12). The single low-risk trial [21] yielded MD = −1.60 (95% CI −1.70 to −1.50). The between-subgroup test was significant (*p* < 0.001) (Figure 4).

Recall period specification. Studies without a specified recall period (k = 17) yielded a significant pooled estimate (MD = −1.58; *p* < 0.001), whereas studies specifying 24-h recall (k = 3, Li 2024 [21], Zhang 2019 [45], Sun 2020 [56]) did not (MD = −1.09; *p* = 0.070). The between-subgroup difference was not significant (Q = 1.99; *p* = 0.16).

Intervention modality. Pooled estimates favored acupuncture across all modality strata, with MDs ranging from −0.59 to −1.75. Heterogeneity was substantial in both MA subgroups (I^2^ = 84–93%) but negligible for EA combined with CBZ (k = 2; I^2^ = 0%).

Maximum carbamazepine dose. Estimates were similar between the lower-dose (≤0.6 g/day; MD = −1.46) and higher-dose (>0.6 g/day; MD = −1.52) strata, with overlapping confidence intervals and substantial heterogeneity in both.

- Meta-Regression of VAS Pain Intensity

Univariable meta-regression was performed for three candidate moderators (Table S8). Session duration showed a borderline association with smaller VAS reductions (β = 0.026; 95% CI −0.002 to 0.054; *p* = 0.066; R^2^ = 10.5%). Baseline pain severity was not a significant moderator (k = 19; β = 0.051; 95% CI −0.353 to 0.454; *p* = 0.79; R^2^ = 0%; Zheng 2010 [52] excluded for lacking baseline means). Neither weekly treatment frequency (β = −0.120; *p* = 0.277) nor De qi (the characteristic needling sensation elicited during manual acupuncture) requirement (β = −0.147; *p* = 0.718) was a significant moderator. Residual heterogeneity exceeded 90% in all models.

#### 3.4.2. SF-MPQ Pain Quality Outcomes

Six trials reported SF-MPQ outcomes [21,36,37,52,54,55]. Given heterogeneity in instrument versions (SF-MPQ vs. SF-MPQ-2) and inconsistent subscale reporting, formal pooling was conducted only where three or more studies provided extractable data for the same subscale. Directionally consistent effects favoring acupuncture were observed across subscales (k = 3 per subscale; I^2^ = 46–70%). Recall period was specified in only one of the six studies. Results are summarized in Table S9.

### 3.5. Follow-Up Pain Intensity

**Figure 5:**
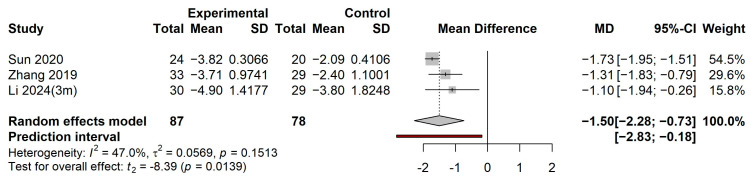
Forest plot of VAS pain intensity at three-month follow-up [21,45,56]. The red horizontal line indicates the prediction interval.

Three-month follow-up. Three trials reported VAS pain intensity at three months after the end of active treatment [21,45,56], comprising 165 participants (87 acupuncture, 78 control), the same three trials that constitute the recall-specified end-of-treatment subgroup in Section 3.4.1. The pooled estimate favored acupuncture (MD = −1.50; 95% CI −2.28 to −0.73; *p* = 0.014). Heterogeneity was moderate (I^2^ = 47.0%; Q = 3.78, *p* = 0.15) (Figure 5). Although the 95% prediction interval (−2.83 to −0.18) did not cross zero, this estimate derived from only three studies, and leave-one-out analysis (Figure S4) showed that the pooled estimate lost significance upon removal of either Zhang 2019 [45] or Li 2024 [21]. The prediction interval is, therefore, unstable and should not be interpreted as evidence of a durable effect.

Six-month follow-up. Two trials reported VAS pain intensity at six months [21,42]. Given the pre-specified threshold of three studies for pooling, results are reported narratively. Pan 2017 [42] observed a significant reduction favoring acupuncture (MD = −1.95; 95% CI −2.40 to −1.50), whereas Li 2024 [21] yielded a smaller, non-significant estimate (MD = −0.70; 95% CI −1.64 to 0.24). Both trials favored acupuncture, but the magnitude of effect differed substantially (MD = −1.95 vs. −0.70), with the larger effect observed in the higher-risk-of-bias trial [42].

### 3.6. Attack Frequency and Duration

Three trials reported weekly attack frequency (Hao 2019, Pan 2017, Li 2024; *n* = 175) [21,41,42]. The pooled estimate did not reach statistical significance (MD = −2.66; 95% CI −14.17 to 8.84; *p* = 0.424; I^2^ = 78.3%). The 95% prediction interval ranged from −1.50 to 0.68 (Figures S5 and S6).

Two trials reported the duration of individual pain attacks [38,46], Both observed reductions favoring acupuncture, but effect sizes diverged substantially: Xing 2021 [38] (SMD = −2.68; 95% CI −3.29 to −2.07) and Huang 2018 [46] (SMD = −0.87; 95% CI −1.39 to −0.35).

### 3.7. Recurrence Rate

Two trials reported recurrence at different time points. Zheng 2010 [52] assessed recurrence at twelve months (RR = 0.555; 95% CI 0.31 to 0.99); Si 2018 [54] assessed recurrence at six months (RR = 0.30; 95% CI 0.09 to 0.99). Both estimates favored acupuncture. As follow-up intervals differed, results were not pooled.

### 3.8. Safety Outcomes

Seven trials reported adverse event data (*n* = 649). One trial [52] did not provide sufficient data for pooling; the remaining six (*n* = 529) were included in the meta-analysis. Acupuncture was associated with a significantly lower risk of adverse events than carbamazepine (RR = 0.31; 95% CI 0.21 to 0.46; I^2^ = 0%). No serious adverse events were reported in either group. Adverse events in the acupuncture groups (2.0–21.2%) were predominantly mild and transient (needle-site pain, hematoma, syncope), whereas those in the carbamazepine groups (13.6–51.5%) were predominantly central nervous system and gastrointestinal disturbances (Table S10).

### 3.9. Quality of Evidence

The certainty of evidence was assessed using the GRADE framework for all five outcomes (Table S11). End-of-treatment VAS pain intensity was rated very low, downgraded for serious risk of bias, inconsistency (I^2^ = 93.1%; prediction interval crossing zero), and indirectness. Three-month follow-up VAS was very low, downgraded for serious risk of bias, indirectness, and imprecision (k = 3; not robust to single-study removal). Attack frequency was very low, with very serious imprecision (95% CI spanning benefit and harm). Recurrence rate was very low owing to serious risk of bias, indirectness, and imprecision from two unpooled estimates. Adverse events were rated very low, downgraded for serious risk of bias due to open-label designs and for serious publication bias concern, given that 17 of 23 trials did not report adverse event data.

## 4. Discussion

This review extends prior syntheses in two directions. First, risk-of-bias stratification reveals that the pooled end-of-treatment analgesic estimate does not reach statistical significance in methodologically stronger trials, and recall period specification was pervasively absent, a measurement limitation whose consequences are amplified in a condition defined by spontaneous remission. Second, this is the first synthesis to examine whether analgesic effects persist after treatment cessation, the outcome dimension that the TRINCOS Delphi consensus ranked as patients’ highest priority [18]. At three-month follow-up, heterogeneity fell substantially and all contributing studies favored acupuncture, though this finding was sensitive to single-study removal and shares the recall period limitation present throughout the evidence base.

### 4.1. Evidential Constraints on End-of-Treatment Pain Intensity Estimates

The pooled VAS estimate favors acupuncture over control interventions by 1.49 points on a 0 to 10 scale. In neuropathic pain trials, the commonly applied minimum clinically important difference for VAS ranges from 1.0 to 1.5 points [57]. The point estimate approaches but does not reach the upper threshold. Among the 20 VAS-reporting trials, pre-treatment pain intensity ranged from 5.12 to 8.23; a 1.49-point between-group difference corresponds to 18 to 29% of baseline, placing it at the boundary between minimally and moderately important change under the Initiative on Methods, Measurement, and Pain Assessment in Clinical Trials (IMMPACT) percentage framework [58]. The prediction interval (−3.09 to 0.02) includes values below all established MCID thresholds, meaning that in a future comparable trial the true effect could range from substantial benefit to a magnitude indistinguishable from no clinical benefit.

Risk-of-bias stratification reveals that statistical significance was confined to the high-risk subgroup (k = 16; *p* < 0.001). The some-concerns subgroup did not reach significance (k = 3; *p* = 0.12), and the single low-risk trial could not contribute to a pooled test. Point estimates were numerically similar across strata, so the difference in significance does not indicate a smaller effect in the stronger trials. It is better explained by two features of the some-concerns subgroup. The subgroup contained only three trials, which limits statistical power. Within-subgroup heterogeneity was also extreme (I^2^ = 98.4%), which widens the confidence interval under the random-effects model. A post-hoc analysis pooling the low-risk and some-concerns trials together (k = 4) yielded a significant estimate in the same direction (MD = −1.37; 95% CI −2.48 to −0.26; *p* = 0.030). This estimate was not robust, as its prediction interval crossed zero (−3.82 to 1.09) and residual heterogeneity remained near 98%. The better-conducted trials, therefore, point in the same direction as the full set, but they do not provide a precise effect estimate.

Subgroup analyses by intervention modality and carbamazepine dose returned overlapping estimates, and univariable meta-regression identified no significant moderator. The concordance across carbamazepine dose subgroups (≤0.6 g vs. >0.6 g) also argues against drug toxicity inflating comparator-arm pain ratings as a driver of the pooled effect, although the open-label design means this comparison remains distinct from a placebo-controlled one. Baseline pain severity, examined across 19 trials, accounted for none of the between-study variance (R^2^ = 0%), and residual heterogeneity remained above 90% in every model. The inconsistency across trials is, therefore, not explained by the candidate moderators tested, and the pooled estimate should be read with corresponding caution.

Approximately two-thirds of TN patients follow a relapsing-remitting course, with spontaneous remissions lasting months to years [6,7]. The median active period is approximately 50 days [5]; remissions can, therefore, begin at any point during a four-week treatment course. Recall period was unspecified in over 85% of VAS outcomes, and no trial documented whether participants were assessed during an active attack or a spontaneous remission. Under these conditions, a pain score at treatment end may reflect the onset of a spontaneous remission rather than a treatment response. In a neuropathic pain trial, weekly recall ratings required more than twice the sample size to detect the same treatment effect as daily ratings of the same construct [59], because retrospective reconstruction inflates measurement noise. TN amplifies this inflation: paroxysmal attacks average 8.5 on a 0 to 10 NRS [60], while remission produces complete absence of pain, so a single retrospective score oscillates between two extremes depending on assessment timing alone. Among the sources of bias that distinguish higher-risk from lower-risk trials in this set, uncontrolled outcome measurement is the one with consequences specific to TN: in a stable chronic pain condition, an unspecified recall period introduces imprecision; in a relapsing-remitting condition, it introduces directional error whose sign depends on disease phase at assessment.

The adverse event profile, by contrast, is methodologically robust: the pooled risk ratio was precise and homogeneous (RR = 0.31; I^2^ = 0%), though 17 of 23 trials did not report adverse events and selective non-reporting cannot be excluded. Twenty-two of 23 trials used active pharmacological comparators without sham controls. In individual-patient data analyses of acupuncture for chronic pain, the effect versus sham is consistently smaller than versus no-acupuncture controls [61]. The single sham-controlled trial produced a point estimate indistinguishable from the overall pool, but one trial cannot establish that this convergence would hold across the remaining 22 uncontrolled comparisons [21]. With almost no sham controls, the observed effect could be attributable to non-specific placebo response, patient expectation, or the natural history of a relapsing-remitting condition. No efficacy conclusion can be drawn from the end-of-treatment data on the current evidence base. Whether the analgesic advantage persists beyond the treatment window is a more direct test, and one aligned with the patient priority identified by the TRINCOS consensus [18].

### 4.2. Exploratory Signal of Durable Analgesic Effects

At three months post-treatment, the pooled VAS estimate was comparable in magnitude to the end-of-treatment value (MD = −1.50; *p* = 0.014), while heterogeneity fell substantially (I^2^ = 47% vs. 93%). Leave-one-out sensitivity analysis showed that this finding was not robust: removal of Zhang 2019 [45] or Li 2024 [21] rendered the pooled estimate non-significant (*p* = 0.12 and *p* = 0.08, respectively). The statistical conclusion, therefore, depends on the joint contribution of all three studies, and the pool shares the same recall period limitations identified for end-of-treatment scores.

Recurrence rate offers a complementary line of evidence that is free of the recall period limitation. Si 2018 reported a risk ratio of 0.30 at six months [54]; Zheng 2010 reported 0.555 at twelve months [52]. The attenuation from six to twelve months is consistent with an effect that diminishes over time but had not disappeared at last follow-up. Both estimates favored acupuncture, aligning directionally with the three-month VAS finding across independent constructs, time points, and study samples. Each line of evidence is individually fragile. The VAS pool is sensitive to single-study removal. The two recurrence trials used different follow-up intervals (six and twelve months), and neither used blinded outcome assessment for recurrence. These data are hypothesis-generating. They do not establish that acupuncture modifies the course of the disease. In chronic musculoskeletal pain, an individual patient data meta-analysis of 39 sham-controlled trials (*n* = 20,827) estimated that approximately 85% of the acupuncture analgesic effect was retained at 12 months [62]. A Cochrane review reported comparable persistence for episodic migraine prevention [63]. Both syntheses were based on adequately blinded, sham-controlled designs with prospective follow-up. The present data are consistent with the possibility that a similar pattern exists in paroxysmal neuropathic pain, but the volume (k = 3), robustness, and measurement quality of the available evidence do not permit an analogous conclusion.

### 4.3. Clinical Positioning

The adverse event profile favoring acupuncture (RR = 0.31; I^2^ = 0%) is the most methodologically secure finding in this review. The favorable adverse event profile, combined with the exploratory finding of analgesic persistence at follow-up, suggests that acupuncture may warrant further investigation as a potential adjunctive intervention for patients in whom dose-limiting carbamazepine toxicity constrains pharmacological management. This does not support its routine clinical adoption on the current evidence. The only factorial trial in this literature found that electroacupuncture combined with low-dose carbamazepine (300 mg/day) produced larger VAS reductions than either intervention alone, with the analgesic advantage persisting through 28 weeks of follow-up; rescue medication use was significantly lower in the electroacupuncture groups at that time point (*p* = 0.011) [21]. These data provide direct, albeit single-trial, evidence that adjunctive acupuncture may permit effective analgesia at a reduced pharmacological burden.

When medical management fails, the established alternatives are surgical or radiosurgical, including microvascular decompression, percutaneous rhizotomy, and stereotactic radiosurgery [12]. Radiosurgery in particular offers pain relief with a low adverse event burden, so a low event rate alone does not distinguish acupuncture from these options [64]. A direct comparison with these procedures was outside the scope of this review, which was restricted to trials of acupuncture against non-acupuncture comparators. Acupuncture is therefore best positioned as a possible adjunct for drug-intolerant patients, not as an alternative to definitive procedural treatment.

A four-week treatment course whose analgesic effect does not attenuate at three months represents a favorable resource profile for primary care. Should confirmatory trials replicate the recurrence reductions observed in this review, the interval between follow-up visits could be extended for patients who respond.

Confirmatory trials should meet several minimum design standards. Sham controls are needed to separate specific from non-specific analgesic effects. Daily pain diaries, as implemented in the erenumab trial for TN [65], would replace retrospective VAS scores with real-time recordings and eliminate the recall period problem that pervades the existing literature. Each assessment should document whether the participant is in an active attack or remission phase, so that disease-cycle confounding can be measured rather than ignored. Follow-up pain intensity at three, six, and twelve months should serve as a co-primary rather than secondary endpoint, reflecting the patient priority established by the TRINCOS consensus. Attack frequency warrants inclusion as a secondary endpoint, given that patients in the same consensus process rated trigger sensitivity as important [18]. Depression and anxiety, which occur at elevated rates in TN [66], should be measured with validated instruments alongside pain and functional outcomes.

### 4.4. Limitations

All included trials were conducted in China, and Chinese acupuncture trials report positive results at higher rates than trials elsewhere [67]. Egger’s test showed no significant asymmetry, but its power is limited at k = 20, so publication bias cannot be excluded. Because management pathways for trigeminal neuralgia differ across regions, our comparisons and conclusions may have reduced relevance outside this setting. Restriction to English and Chinese publications and inconsistent diagnostic criteria add further unmeasured heterogeneity. Second, 22 of 23 trials used active pharmacological comparators without sham controls, leaving non-specific effects unquantified. Third, follow-up evidence is limited in both volume and measurement quality: the three-month VAS estimate derived from only three studies and lost significance on removal of any single contributor; the recurrence data came from two single trials at different time points; and the recall period problem in end-of-treatment scores is equally present at follow-up. These constraints render all follow-up findings exploratory and limit precision at every time point. Fourth, key clinical moderators were inconsistently reported and could not be examined, including baseline severity, trigeminal branch distribution, attack-remission phase, classic (Type I) versus mixed (Type II) presentation, disease duration, and prior invasive treatment. Pooling manual and electroacupuncture, and trials with and without concurrent medication, may further obscure modality-specific effects. Finally, we did not contact original authors for unreported recall periods or missing data, since our objective was to characterize the quality of the published evidence as it currently stands.

## 5. Conclusions

End-of-treatment pain intensity estimates for acupuncture in trigeminal neuralgia are compromised by risk-of-bias dependence, pervasive recall period omission, and near-absence of sham controls. By stratifying on risk of bias, auditing recall period reporting, and examining post-treatment follow-up for the first time, this review identifies why the existing evidence cannot yet support an efficacy conclusion. The clearest positive finding is that acupuncture was associated with significantly fewer adverse events than carbamazepine, with no heterogeneity across trials. Follow-up data showed a directionally consistent but statistically fragile signal that analgesia may persist beyond the treatment period. This signal is exploratory and hypothesis-generating, as it was not robust to single-study removal. With only one sham-controlled trial, the current evidence cannot separate specific analgesic effects from non-specific placebo response, and it does not support routine clinical adoption. The favorable safety profile and the directional follow-up signal together justify rigorous confirmatory trials, with sham controls, prospective pain diaries, documented disease phase, and follow-up as a co-primary endpoint.

## Figures and Tables

**Table 1:** Summary of the characteristics of the included studies.

Study (Year)	Overall Age (Mean ± SD)	Sample Size (T/C)	Sex (M/F)		Intervention		Endpoint (Weeks)	Outcome	Ref.
			T	C	T	C			
1. Wang (2024)	44.30 ± 5.01	34/34	7/27	9/25	MA + CBZ	CBZ (0.3–0.6 g daily)	2	(1) Pain (VAS)	[35]
2. Cao (2024)	52.17 ± 5.19	25/25	12/13	10/15	MA + CBZ	CBZ (0.6–1.6 g daily)	4	(1) Pain (SF-MPQ)	[36]
3. Hou (2023)	57.33 ± 3.57	48/48	17/31	19/29	MA + CBZ	CBZ (0.2–1.5 g daily)	12	(1) Pain (SF-MPQ) (2) AE	[37]
4. Xing (2021)	55.03 ± 3.18	40/40	18/22	16/24	MA + CBZ	CBZ (0.4–0.8 g daily)	8	(1) Pain (VAS) PD	[38]
5. Tan (2021)	55.2 ± 6.3	33/33	15/18	14/19	MA + CBZ	CBZ (0.3–0.6 g daily)	4	(1) Pain (VAS)	[39]
6. Niu (2021)	56.43 ± 3.25	36/36	16/20	15/21	MA + CBZ	CBZ (0.3 g daily)	4	(1) Pain (VAS)	[40]
7. Hao (2019)	45.6 ± 3.4	27/27	12/15	13/14	MA + CBZ	CBZ (0.2–1.2 g daily)	4	(1) Pain (VAS) AF	[41]
8. Pan (2017)	54 ± 11	31/30	13/18	12/18	MA + CBZ	CBZ (0.2–1.2 g daily)	4	(1) Pain (VAS) (2) FU-VAS (6 m) (3) AF	[42]
9. Chen (2021)	43.28 ± 6.43	40/40	16/24	18/22	MA	CBZ (0.2–1.0 g daily)	4	(1) Pain (VAS)	[43]
10. Liu (2019)	48.72 ± 5.27	44/44	17/27	19/25	MA	CBZ (0.2–1.0 g daily)	4	(1) Pain (VAS)	[44]
11. Zhang (2019)	47.3 ± 5.7	34/30	12/22	11/19	MA	CBZ (0.3–0.6 g daily)	4	(1) Pain (VAS) (2) FU-VAS (3 m) (3) AE	[45]
12. Huang (2018)	43.64 ± 5.47	32/31	12/21	11/20	MA	CBZ (0.2–1.0 g daily)	4	(1) Pain (VAS) (2) PD (3) AE	[46]
13. Yang (2018)	42.6 ± 5.7	50/50	23/27	22/28	MA	CBZ (0.2–1.2 g daily)	4	(1) Pain (VAS)	[47]
14. Wei (2018)	45.74 ± 7.54	31/31	18/13	19/12	MA	CBZ (0.6 g daily)	4	(1) Pain (VAS)	[48]
15. Xiao (2016)	54.2 ± 11.5	50/50	13/37	15/35	MA	CBZ (0.2–0.6 g daily)	4	(1) Pain (VAS)	[49]
16. Meng (2014)	54.2 ± 2.5	50/50	30/20	26/24	MA	CBZ (0.2–1.2 g daily)	4	(1) Pain (VAS) (2) AE	[50]
17. Wang (2013)	54.54 ± 2.3	20/20	3/17	5/15	MA	CBZ (0.2–1.2 g daily)	6	(1) Pain (VAS)	[51]
18. Zheng (2010)	52 ± 14.75	60/60	28/32	27/33	MA	CBZ (0.45 g daily)	4	(1) Pain (VAS) (2) RR (12 m)	[52]
19. Di (2021)	57.27 ± 6.28	30/30	13/17	12/18	MA	CBZ (0.6–1.6 g daily)	4	(1) Pain (VAS) (2) Pain (SF-MPQ) (3) AE	[53]
20. Li (2024)	54.2 ± 18.8	30/30	15/15	11/19	EA + CBZ	SEA + CBZ	4	(1) Pain (VAS) (2) Pain (SF-MPQ) (3) FU-VAS (3 m, 6 m) (4) AF (5) AE	[21]
21. Si (2018)	56.36 ± 7.56	33/33	12/21	13/20	EA + CBZ	CBZ (0.3–0.6 g daily)	4	(1) Pain (VAS) (2) Pain (SF-MPQ) (3) RR (6 m) (4) AE	[54]
22. Yin (2018)	62.37 ± 5.41	62/62	25/37	27/35	EA + CBZ	CBZ (0.2–1.2 g daily)	4	(1) Pain (SF-MPQ)	[55]
23. Sun (2020)	58 ± 10	24/20	8/16	7/13	EA	CBZ (0.3 g daily)	4	(1) Pain (VAS) (2) FU-VAS (3 m)	[56]

T, treatment group; C, control group; M/F, male/female; MA, manual acupuncture; EA, electroacupuncture; SEA, sham electroacupuncture; CBZ, carbamazepine; VAS, Visual Analog Scale; SF-MPQ, Short-Form McGill Pain Questionnaire; SF-MPQ-2, Short-Form McGill Pain Questionnaire-2; FU-VAS, follow-up Visual Analog Scale pain intensity; AF, weekly attack frequency; PD, duration of individual pain attacks; RR, recurrence rate. Parenthetical values denote follow-up time point in months; AE, adverse events.

**Table 2:** Subgroup meta-analysis of acupuncture effects on VAS pain intensity.

Analysis Subgroup	No. of RCTs (k)	MD	95% CI	*p* Value	Heterogeneity (I^2^,%)	95% PI
Main analysis (Overall)	20	−1.49	[−1.81; −1.16]	<0.001	93.1%	[−3.09; 0.02]
Overall Risk of Bias Level						
High risk of bias	16	−1.53	[−1.92; −1.14]	<0.001	87.2%	[−3.02; −0.04]
Some concerns + Low (pooled)	4	−1.37	[−2.48; −0.26]	0.030	97.8%	[−3.82; 1.09]
Some concerns	3	−1.28	[−3.35; 0.79]	0.117	98.4%	[−5.41; 2.85]
Low risk of bias	1	−1.60	[−1.70; −1.50]	-	-	-
Recall Period Specification						
Not specified	17	−1.58	[−1.95; −1.21]	<0.001	91.6%	[−3.05; −0.10]
Recall period specified	3	−1.09	[−2.39; 0.22]	0.070	95.9%	[−3.67; 1.50]
Intervention Modality						
MA + CBZ vs. CBZ	6	−1.18	[−2.01; −0.36]	0.014	93.2%	[−3.29; 0.92]
MA vs. CBZ	11	−1.75	[−2.17; −1.33]	<0.001	84.4%	[−3.06; −0.44]
EA + CBZ vs. CBZ	2	−1.60	[−1.70; −1.50]	<0.001	0%	-
EA vs. CBZ	1	−0.59	[−0.86; −0.31]	<0.05	-	-
Maximum Carbamazepine Dose						
≤0.6 g/d	10	−1.46	[−1.93; −0.99]	<0.001	95.1%	[−2.91; −0.02]
>0.6 g/d	10	−1.52	[−2.09; −0.96]	<0.001	89.1%	[−3.31; 0.26]

MD, mean difference; CI, confidence interval; I^2^, inconsistency statistic; k, number of randomized controlled trials; PI, prediction interval; MA, manual acupuncture; EA, electroacupuncture; CBZ, carbamazepine. Pooled effect estimates were calculated using a random-effects model.

## Data Availability

All data generated or analyzed during this study are included in this published article and its Supplementary Information files, further inquiries can be directed to the corresponding author.

## Supplementary Materials

The following supporting information can be downloaded at: https://www.mdpi.com/article/10.3390/healthcare14131926/s1, Table S1: Full search terms. Table S2: Inclusion and Exclusion Criteria for Study Selection. Table S3: Descriptions of the acupuncture interventions. Table S4: Reporting quality and standardization of VAS for pain intensity in acupuncture trials for trigeminal neuralgia. Table S5: Reporting characteristics of VAS-based pain outcomes (*n* = 20). Table S6: Reporting quality and standardization of the SF-MPQ in acupuncture trials for trigeminal neuralgia. Table S7: Sensitivity analysis of end-of-treatment VAS pain intensity under alternative imputation correlation coefficients. Table S8: Univariable meta-regression analyses of potential moderators for the effect of acupuncture on VAS pain intensity in trigeminal neuralgia. Table S9: Meta-analysis of acupuncture effects on SF-MPQ pain outcomes. Table S10: Summary of Adverse Events Reported in Included Studies. Table S11: Summary of Adverse Events Reported in Included Studies. Figure S1: Leave-one-out analysis for the effect on VAS scores. Figure S2: Funnel plot assessing publication bias for VAS pain intensity outcomes. Figure S3: Forest plot of VAS pain intensity excluding the single sham-controlled trial. Figure S4: Leave-one-out analysis for the effect on VAS scores at 3-month follow-up. Figure S5: Forest plot of weekly attack frequency. Figure S6: Leave-one-out analysis of weekly attack frequency.
