# Supplementary material for: Does Acupuncture Produce Durable Analgesia in Trigeminal Neuralgia? An Updated Systematic Review and Meta-Analysis
Source: Healthcare (Basel). 2026 Jul 1;14(13):1926. doi: 10.3390/healthcare14131926 (PMC13360922; doi:10.3390/healthcare14131926)

Table S1. Full search terms

**PubMed**	#1 "Trigeminal Neuralgia"[MeSH Terms]<br>#2 "trigeminal neuralgia"[Title/Abstract] OR "primary trigeminal neuralgia"[Title/Abstract]<br>OR "idiopathic trigeminal neuralgia"[Title/Abstract]<br>OR "classical trigeminal neuralgia"[Title/Abstract]<br>OR "tic douloureux"[Title/Abstract]<br>#3 #1 OR #2<br>#4 "Acupuncture Therapy"[MeSH Terms] OR "Electroacupuncture"[MeSH Terms]<br>OR "Acupuncture Points"[MeSH Terms]<br>#5 "acupuncture"[Title/Abstract] OR "electroacupuncture"[Title/Abstract]<br>OR "manual acupuncture"[Title/Abstract] OR "body acupuncture"[Title/Abstract]<br>OR "acupoint*"[Title/Abstract] OR "meridian*"[Title/Abstract]<br>OR "needling"[Title/Abstract]<br>#6 #4 OR #5<br>#7 "Randomized Controlled Trial"[Publication Type]<br>OR "Clinical Trial"[Publication Type]<br>OR "Random Allocation"[MeSH Terms]<br>OR "Randomized Controlled Trials as Topic"[MeSH Terms]<br>OR "random*"[Title/Abstract]<br>OR ("clinical"[Title/Abstract] AND "trial"[Title/Abstract])<br>#8 #3 AND #6 AND #7
**Embase**	#1 'trigeminal neuralgia'/exp/mj<br>#2 'primary trigeminal neuralgia':ti,ab,kw<br>OR 'idiopathic trigeminal neuralgia':ti,ab,kw

	OR 'classical trigeminal neuralgia':ti,ab,kw<br>OR 'tic douloureux':ti,ab,kw<br>#3 #1 OR #2<br>#4 'acupuncture therapy'/exp/mj OR 'electroacupuncture'/exp/mj<br>OR 'acupuncture point'/exp/mj<br>#5 'acupuncture':ti,ab,kw OR 'electroacupuncture':ti,ab,kw<br>OR 'manual acupuncture':ti,ab,kw OR 'body acupuncture':ti,ab,kw<br>OR 'acupoint*':ti,ab,kw OR 'meridian*':ti,ab,kw<br>OR 'needling':ti,ab,kw<br>#6 #4 OR #5<br>#7 'randomized controlled trial'/exp/mj<br>OR 'controlled clinical trial'/exp/mj<br>OR 'random allocation'/exp/mj<br>OR 'clinical trial'/exp/mj<br>#8 'randomized controlled trial':ti,ab,kw<br>OR 'randomised controlled trial':ti,ab,kw<br>OR 'random*':ti,ab,kw<br>OR ('clinical':ti,ab,kw AND 'trial':ti,ab,kw)<br>#9 #7 OR #8<br>#10 #3 AND #6 AND #9
**Web of Science**	#1 TS=("trigeminal neuralgia" OR "primary trigeminal neuralgia" OR "idiopathic trigeminal neuralgia" OR "classical trigeminal neuralgia" OR "tic douloureux")<br>#2 TS=(acupuncture OR electroacupuncture OR "manual acupuncture" OR "body acupuncture" OR acupoint* OR meridian* OR needling)<br>#3 TS=("randomized controlled trial" OR "randomised controlled trial" OR "clinical trial" OR random*)

	#4 #1 AND #2 AND #3
**Chinese Biomedical Literature Database (CBM)**	#1 Subject heading: "Trigeminal Neuralgia" [Exploded]<br>#2 "Trigeminal Neuralgia" [Title/Abstract] OR "Primary Trigeminal Neuralgia" [Title/Abstract] OR "Idiopathic Trigeminal Neuralgia" [Title/Abstract]<br>#3 #1 OR #2<br>#4 Subject heading: "Acupuncture Therapy" [Exploded] OR "Electroacupuncture" [Exploded] OR "Acupuncture Points" [Exploded]<br>#5 "Acupuncture" [Title/Abstract] OR "Acupuncture Therapy" [Title/Abstract] OR "Electroacupuncture" [Title/Abstract] OR "Manual Acupuncture" [Title/Abstract] OR "Acupoint" [Title/Abstract] OR "Acupuncture Point" [Title/Abstract]<br>#6 #4 OR #5<br>#7 Subject heading: "Randomized Controlled Trial" [Exploded]<br>#8 "Randomized Controlled Trial" [Title/Abstract] OR "Randomized Clinical Trial" [Title/Abstract] OR "Random Allocation" [Title/Abstract] OR "Randomized" [Title/Abstract] OR "Random" [Title/Abstract]<br>#9 #7 OR #8<br>#10 #3 AND #6 AND #9
**China National Knowledge Infrastructure (CNKI)**	#1 SU = ("Trigeminal Neuralgia" + "Primary Trigeminal Neuralgia" + "Idiopathic Trigeminal Neuralgia")<br>#2 SU = ("Acupuncture Therapy" + "Acupuncture" + "Acupuncture Treatment" + "Electroacupuncture" + "Acupoint Needling")<br>#3 SU = ("Randomized Controlled Trial" + "Randomized Clinical Trial") OR TI = ("Random")<br>#4 #1 AND #2 AND #3
**Wan-Fang & Chinese Scientific Journal Database**	("Trigeminal Neuralgia" OR "Primary Trigeminal Neuralgia" OR "Idiopathic Trigeminal Neuralgia") AND ("Acupuncture" OR "Acupuncture Therapy" OR

**(VIP)**	"Electroacupuncture" OR "Acupoint Needling" OR "Manual Acupuncture") AND ("Randomized Controlled Trial" OR "Randomized Clinical Trial" OR "Random Allocation" OR "Random")

Table S2. Inclusion and Exclusion Criteria for Study Selection

PICOS Element	Inclusion Criteria	Exclusion Criteria
**Population**	a) Adult patients (≥18 years) with clinical diagnosis of trigeminal neuralgia<br>b) Classical or idiopathic trigeminal neuralgia; secondary TN acceptable only if data for classical TN patients can be clearly extracted separately<br>c) No restrictions on disease duration or baseline pain severity	a) Studies including patients with other facial pain conditions (postherpetic neuralgia, atypical facial pain, temporomandibular disorders, dental pain) without separate TN data<br>b) Studies with <20 participants per treatment arm<br>c) Animal or in vitro studies
**Intervention**	a) Manual acupuncture (MA)<br>b) Electroacupuncture (EA)<br>c) Combination of MA and EA<br>d) Acupuncture as monotherapy or adjunct therapy<br>e) Treatment duration ≥2 weeks or ≥4 sessions	a) Studies investigating other Traditional Chinese Medicine modalities as primary intervention (herbal medicine, moxibustion, cupping) without acupuncture<br>b) Studies comparing different acupuncture techniques only, without conventional treatment or sham control<br>c) Invasive procedures other than acupuncture (nerve blocks, radiofrequency ablation, surgical interventions)
**Comparison**	a) Conventional pharmacological treatment (carbamazepine, gabapentinoids, other antiepileptic drugs, analgesics)<br>b) Sham acupuncture<br>c) Placebo	a) Studies comparing acupuncture with non-pharmacological interventions only (e.g., physiotherapy, psychological therapy) without conventional treatment control

**Outcomes**	**Primary outcome:**<br>Pain intensity measured by Visual Analogue Scale (VAS) or Short-Form McGill Pain Questionnaire (SF-MPQ) Secondary outcomes: a) Pain intensity at follow-up (assessed ≥1 month after end of treatment)<br>**Secondary outcomes:**<br>a) Pain intensity at follow-up (assessed ≥1 month after end of treatment)<br>b) Weekly attack frequency<br>c) Duration of individual pain attacks<br>d) Recurrence rate<br>e) Safety: Adverse events (AEs), serious adverse events (SAEs)	a) Studies reporting only physiological/objective measures (neuroimaging, electrophysiology, biomarkers) without patient-reported pain outcomes
**Study Design**	a) Peer-reviewed publications of Randomized controlled trials (RCTs)<br>b) Parallel-group or crossover design<br>c) Pilot and feasibility studies meeting other criteria	a) Case reports, case series, cohort studies, case-control studies, before-after studies<br>b) Ongoing studies without published results<br>c) Study protocols without outcome data<br>d) Conference abstracts without full-text publications<br>e) Duplicate publications (most complete/recent version retained)<br>f) Retrospective studies<br>g) Systematic reviews and meta-analyses*
**Publication Characteristics**	a) Published in English or Chinese<br>b) No restrictions on publication date	

*Reference lists of systematic reviews and meta-analyses were screened for potentially eligible primary studies.

Table S3. Descriptions of the acupuncture interventions

	Study (Year)	Acupuncture Point formula	Response sought	Acupuncture treatment variables				
				Minutes per session (min.)	Weekly treatment frequency (sessions per week)	Treatment duration (week)	Total number of treatment sessions	Cumulative treatment time (min.)
**1.**	Wang (2024)	Main acupoints: BL17, LI4, LR3, Matching acupoints: GB14, BL2, EX-HN4, SI18, ST7, ST6	De qi	30	7	2	14	420
**2.**	Cao (2024)	ST7, TE17	Not mentioned	30	7	4	28	840
**3.**	Hou (2023)	LI4, LR3, ST7	Pain or redness	-	7	12	90	-
**4.**	Xing (2021)	BL2, ST2, ST4, ST7, LI4, LR3, ST44	De qi	30	7	8	56	1680
**5.**	Tan (2021)	EX-HN4, ST7, ST6, ST2, CV24, PC6, LI4	Not mentioned	30	7	4	30	900
**6.**	Niu (2021)	GB12, EX-HN3, GV24, GB20, TE5, LI4, BL10	De qi	30	7	4	30	900
**7.**	Hao (2019)	Main acupoints: GB7, BL2, ST2, EX-HN Matching acupoints: ST7, EX-HN5, GB20, GV20, LI4,	De qi	30	7	4	30	900
**8.**	Pan (2017)	Main acupoints: LI20, EX-HN22, ST36 Matching acupoints: ST2, ST4, ST6, ST7, ST3, ST44, LI4, BL2, GB14, SI18, CV24, LR3	De qi	30	6	4	24	720
**9.**	Chen (2021)	Main acupoints: EX-HN5, GB20, LI4, Matching acupoints: DU26, GB14. ST2, ST7	De qi	25	7	4	28	700
**10.**	Di (2021)	GV20, CV17, LR14, CV6, LI4, LR3, ST44	De qi	30	5	4	20	600
**11.**	Liu (2019)	Main acupoints: GB20, GV24, LI4, EX-HN3, BL10. GB12, GV26 Matching acupoints: GB14, LI7, GV20, ST7, ST2,BL2, TE23, EX-HN5, ST4	De qi	20	7	4	28	560
**12.**	Zhang (2019)	Main acupoints: SI8, GB34, ST40 Matching acupoints: EX-HN4, ST2, ST7,	De qi	40	6	4	24	960
**13.**	Huang (2018)	Main acupoints: EX-HN5, ST7, GB20, LI4, Matching acupoints: LI7, ST6, EX-HN3,	De qi	30	7	4	30	900

		GV20						
**14.**	Yang (2018)	Main acupoints: EX-HN3, DU26, DU24, GB12, BL10, GB20, L14, SJ5, Matching acupoints: GB14, BL2, GB15, SJ23, EX-HN5	Not mentioned	30	7	4	30	900
**15.**	Wei (2018)	Main acupoints: GV24, GV26, EX-HN3, DU26, DU24, GB12, BL10, GB20, SJ5, LI14 Matching acupoints: ST8, GB14, BL2, GB15, TE23, EX-HN5, ST2, GB3, SI18, ST5, ST7, ST4	De qi	20	7	4	30	900
**16.**	Xiao (2016)	LI4, SP10, BL17, GB20, GB14, TE17, EX-HN5, ST8, TE23, ST2, ST7, LI20, SI18, ST6, ST4, CV24	De qi	40	6	4	24	960
**17.**	Meng (2014)	Main acupoints: EX-HN5, Matching acupoints: LI4, LI7, GB4, GB20, ST7, ST3, EX-HN3, GV23, GV20	De qi	30	5	4	20	600
**18.**	Wang (2013)	Main acupoints: EX-HN5, Matching acupoints: GV23, GV20, EX-HN3, ST4, ST7, GB20, GB8, LI7, LI4	De qi	30	5	6	30	900
**19.**	Zheng (2010)	Main acupoints: ST9 Matching acupoints: GB14, BL2, EX-HN4, ST2, LI20, ST4, ST6, RN24	De qi	30	5	5.6	30	900
**20.**	Li(2024)	Main acupoints: ST2, ST4, ST7 Matching acupoints: GB1, SI18, ST6	stronger sensation	60	3	4	12	720
**21.**	Si (2018)	EX-HN5, ST2, ST7, ST6, CV24, GB20	De qi	20-30	7	4	30	600-900
**22.**	Yin (2018)	EX-HN5, ST8, ST2, ST7, SI18, ST6, CV24, GB14, GB20, LI4, LR3	De qi	30	7	4	28	840
**23.**	Sun (2020)	ST2, ST4, ST7, GB1, SI18, ST6, LI4, LR3, SJ5, SP6, BL2	stronger sensation	60	3	4	12	720

Table S4. Reporting quality and standardization of VAS for pain intensity in acupuncture trials for trigeminal neuralgia

Study (Year)	Intervention		Patient self-report? (Y/N/Unclear)	Assessment Timeframe	Scale range	Anchor labels reported (Y/N)	Pain question wording reported? (Y/N)	Reference Cited (Y/N)
	T	C						
**Wang (2024)**	MA+ CBZ	CBZ (0.3-0.6g daily)	Y	NS	0-10	Y	N	Y
**Xing (2021)**	MA+ CBZ	CBZ (0.4-0.8g daily)	Y	NS	0-10	Y	N	N
**Tan (2021)**	MA+ CBZ	CBZ (0.3-0.6g daily)	Unclear	NS	0-9[#]	Y	Y	Y
**Niu (2021)**	MA+ CBZ	CBZ (0.3g daily)	Unclear	NS	0-10	Y	Y	N
**Hao (2019)**	MA+ CBZ	CBZ (0.2–1.2g daily)	Unclear	NS	NS	N	N	N
**Pan (2017)**	MA+ CBZ	CBZ (0.2-1.2g daily)	Y	NS	0-10	Y	Y	Y
**Chen (2021)**	MA	CBZ (0.2-1.0g daily)	Unclear	NS	0-10	Y	N	Y
**Di (2021)**	MA	CBZ (0.6-1.6g daily)	Unclear	NS	NS	N	N	N
**Liu (2019)**	MA	CBZ (0.2-1.0g daily)	Unclear	NS	0-10	Y	N	Y
**Zhang (2019)**	MA	CBZ (0.3-0.6g daily)	Y	average 24 h pain	0-10	Y	Y	Y
**Huang (2018)**	MA	CBZ (0.2-1.0g daily)	Unclear	NS	0-10	Y	N	Y
**Yang (2018)**	MA	CBZ (0.2-1.2g daily)	Unclear	NS	NS	N	N	N
**Wei (2018)**	MA	CBZ (0.6g daily)	Unclear	NS	NS	N	N	N
**Xiao (2016)**	MA	CBZ (0.2-0.6g daily)	Unclear	NS	0-10	Y	N	N
**Meng (2014)**	MA	CBZ (0.2–1.2g daily)	Y	NS	0-10	Y	Y	N
**Wang (2013)**	MA	CBZ (0.2-1.2g daily)	Y	NS	0-10	Y	Y	Y
**Zheng (2010)**	MA	CBZ (0.45g daily)	Y	NS	0-10	Y	Y	Y
**Li (2024)**	EA+ CBZ	SEA+CBZ (0.3g daily)	Y	average 24 h pain	0-10	Y	Y	Y
**Si (2018)**	EA+ CBZ	CBZ (0.3-0.6g daily)	Y	NS	0-10	Y	Y	Y
**Sun (2020)**	EA	CBZ (0.3g daily)	Y	24h pain	0-10	Y	Y	Y

D4: outcome measurement domain of the Cochrane RoB 2 tool; MA, manual acupuncture; EA, electroacupuncture; CBZ, carbamazepine; SEA, sham electroacupuncture; NS, not specified;

#Tan (2021) used a non-standard 0–9 VAS scale. Reported scores were rescaled to 0–10 (multiplied by 10/9) before inclusion in the pooled analysis.

* Patient self-report indicates explicit description of patient self-administration; Timeframe refers to the recall period (e.g., 24-h average); Scale range denotes numerical limits; Anchor labels indicate verbal descriptors for endpoints; Instrument reference indicates citation of established scales.

Table S5. Reporting characteristics of VAS-based pain outcomes (n = 20)

Reporting item	Category	n (%)
**Patient self-report**	Clearly patient-reported (Y)	8 (40.0)
	Unclear assessor	12 (60.0)
**Assessment Timeframe**	Yes (24 h / average 24 h)	3 (15.0)
	Not specified	17 (85.0)
**Scale range reported**	0–10	13 (65.0)
	0–9 (non-standard)	1 (5.0)
	Not specified	6 (30.0)
**Anchor labels reported**	Yes	13 (65.0)
	No	7 (35.0)
**Pain question wording reported**	Yes	7 (35.0)
	No	13 (65.0)
**Reference cited for VAS**	Yes	9 (45.0)
	No	11 (55.0)

Table S6. **Reporting quality and standardization of the SF-MPQ in acupuncture trials for trigeminal neuralgia**

Study (Year)	Intervention		Instrument Version (scale name)	Reported subscales	Assessment Timeframe	Reference Cited (Y/N)
	T	C				
**Cao (2024)**	MA+ CBZ	CBZ (0.6-1.6g daily)	SF-MPQ-2	PRI-S, PRI-A	NS	Y
**Hou (2023)**	MA+ CBZ	CBZ (0.2-1.5g daily)	SF-MPQ	Total score	NS	Y
**Zheng (2010)**	MA	CBZ (0.45g daily)	SF-MPQ	PPI, PRI-T, PRI-S, PRI-A, VAS	NS	Y
**Li (2024)**	EA+ CBZ	SEA+CBZ	SF-MPQ	PPI, PRI-T,	in the past two weeks	Y
**Si (2018)**	EA+ CBZ	CBZ (0.3-0.6g daily)	SF-MPQ	PPI, PRI-T NWC, VAS, Total score	NS	Y
**Yin (2018)**	EA+ CBZ	CBZ (0.2–1.2g daily)	SF-MPQ	Total score	NS	Y

D4: outcome measurement domain of the Cochrane RoB 2 tool; MA, manual acupuncture; CBZ, carbamazepine; EA, electroacupuncture; NS, not specified; NWC, Number of Words Chosen; PPI, Present Pain Intensity; PRI-A, affective Pain Rating Index; PRI-S, sensory Pain Rating Index; PRI-T, total Pain Rating Index; SEA, sham electroacupuncture; SF-MPQ, Short-Form McGill Pain Questionnaire; SF-MPQ-2, Short-Form McGill Pain Questionnaire-2 .

Table S7. Sensitivity analysis of end-of-treatment VAS pain intensity under alternative imputation correlation coefficients

Correlation coefficient (r)	No. of RCTs (k)	MD	95% CI	P	I^2	95% PI
**0.3**	**20**	−1.40	[−1.89; −0.91]	<0.001	94.1%	[−3.56; 0.75]
**0.5 (primary)**	**20**	−1.49	[−1.81; −1.16]	<0.001	93.1%	[-3.09; 0.02]
**0.7**	**20**	−1.42	[−1.91; −0.94]	<0.001	96.0%	[−3.59; 0.74]

CI, confidence interval; k, number of randomized controlled trials; MD, mean difference; PI, prediction interval; I^2, inconsistency statistic.

*All estimates were derived from random-effects models (REML; Hartung-Knapp-Sidik-Jonkman adjustment). Change-from-baseline standard deviations were imputed assuming a pre-post correlation of r = 0.5 in the primary analysis, in accordance with Cochrane recommendations. Sensitivity analyses using r = 0.3 and r = 0.7 produced materially similar pooled estimates, indicating that the conclusions were robust to the choice of imputation correlation. A negative MD indicates a greater reduction in pain scores in the acupuncture group relative to control.

Table S8. Univariable meta-regression analyses of potential moderators for the effect of acupuncture on VAS pain intensity in trigeminal neuralgia

Potential Moderator	No. of Trials (k)	β	95% CI	p	R^2
**Baseline pain severity**	19*	0.051	−0.353 to 0.454	0.79	0.00
**Minutes per session**	20	0.026	−0.002 to 0.054	0.066	0.11
**Weekly frequency**	20	−0.136	−0.370 to 0.098	0.253	0.19
**De qi requirement**	20	−0.147	−0.944 to 0.65	0.718	0.00

CI, confidence interval; k, number of randomized controlled trials

*Zheng 2010 was excluded, as it reported only between-group differences without baseline means. All analyses were performed using univariable random-effects meta-regression models. The β coefficient represents the change in the MD of VAS scores for every one-unit increase in the moderator. A positive β associated with session duration suggests that

longer sessions may correlate with a smaller reduction in pain intensity. R2 indicates the proportion of between-study variance explained by the moderator.

Table S9. Meta-analysis of acupuncture effects on SF-MPQ pain outcomes

Outcome	No. of RCTs (k)	MD	95% CI	P	I^2	95% PI
**PPI**	3	-0.99	[-1.59; -0.39]	0.0192	46.3%	[-1.94; -0.05]
**PRI-T**	3	-3.44	[-6.37; -0.51]	0.0370	59.2%	[-7.94; 1.05]
**SF-MPQ Total**	3	-6.81	[-10.82; -2.79]	0.0183	70.3%	[-15.79; 2.18]

PPI, Present Pain Intensity; PRI-T, Pain Rating Index total score; k, number of randomized controlled trials; MD, mean difference; CI, confidence interval; PI, prediction interval; I^2, inconsistency statistic

*All pooled estimates were calculated using a random-effects model. PPI (Present Pain Intensity) is scored on a 0–5 scale; PRI-T (Pain Rating Index total score) and SF-MPQ total score are derived from the Short-Form McGill Pain Questionnaire (SF-MPQ). A negative MD indicates a greater reduction in pain scores in the acupuncture group relative to control.

Table S10. Summary of Adverse Events Reported in Included Studies

Study (Year)	Experimental Intervention	n (T/C)	AEs in Experimental Group (type)	AE Incidence, Experimental Group	AEs in Control Group (type)	AE Incidence, Control Group	Serious AEs
**Hou (2023)**	MA + CBZ	48/48	Dizziness (n=1), taste disturbance (n=1), other (n=2)	4/48 (8.33%)	Dizziness (n=3), taste disturbance (n=2), cardiovascular symptoms (n=2), inflammatory skin reaction (n=1), other (n=4)	12/48 (25.00%)	None
**Liu (2019)**	MA	44/44	NR	1/44 (2.27%)	NR	6/44 (13.64%)	None
**Zhang (2019)**	MA	33/29	Fatigue (n=1), dizziness (n=1), drowsiness (n=1)	3/33 (9.1%)	Fatigue (n=2), dizziness (n=1), drowsiness (n=2), gastrointestinal reaction (n=2)	7/29 (24.1%)	None
**Xiao (2016)**	MA	50/50	Needle syncope (n=1)	1/50 (2%)	Gastrointestinal reactions (n=6), mild liver function abnormalities (n=4), rash (n=2)	12/50 (24%)	None
**Zheng (2010)**	MA	60/60	Needle-site pain	NR	NR	NR	None
**Li (2024)**	EA + CBZ	30/30	hematoma (n=3)	3/30(10.0%)	Dizziness (n=6), dermatitis (n=5), palpitations (n=4)	15/30(50.0%)	None
**Si (2018)**	EA + CBZ	33/33	Peripheral facial numbness (n=1), facial numbness (n=1), tinnitus (n=3), herpes zoster infection (n=2)	7/33 (21.21%)	Peripheral facial numbness (n=2), facial numbness (n=4), tinnitus (n=5), herpes zoster infection (n=6)	17/33 (51.51%)	None
**Pooled**	—	542	Needle-site pain; needle syncope; hematoma; dizziness; fatigue; drowsiness; peripheral facial numbness; tinnitus; herpes zoster infection	2.0%–21.21%	Dizziness, drowsiness, fatigue; gastrointestinal reactions; liver function abnormalities; rash, dermatitis; palpitations; taste disturbance; cardiovascular symptoms; tinnitus; herpes zoster infection	13.64%–51.51%	None

AE, adverse event; MA, manual acupuncture; EA, electroacupuncture; SEA, sham electroacupuncture; CBZ, carbamazepine; T, treatment group; C, control group; NR, not reported at individual study level (aggregate incidence range reported across studies). All AEs were classified as mild to moderate in severity. No treatment discontinuations due to AEs were reported in either group. Pooled RR = 0.31 (95% CI 0.21–0.46; $p < 0.001$; $I^2 = 0\%$) favoring acupuncture.

*Pooled RR was calculated from six trials (n=471) with extractable event rates; Zheng (2010) reported AEs narratively without incidence proportions and was therefore excluded from quantitative pooling.

Table S11. Summary of Adverse Events Reported in Included Studies

	Certainty assessment					№ of patients		Effect		Certainty
**Outcome / № of studies / Study design**	**Risk of bias**	**Inconsistency**	**Indirectness**	**Imprecision**	**Other considerations**	**Acupuncture**	**Control**	**Relative (95% CI)**	**Absolute (95% CI)**	
**End-of-treatment VAS pain intensity**										
**20 Randomized**	Serious [a]	Serious [b]	Serious [c]	Not serious	None	779	779	–	MD 1.49 lower (1.81 lower to 1.16 lower)	⊕◯◯◯ VERY LOW
**Follow-up VAS pain intensity (3 months)**										
**3 Randomized**	Serious [d]	Not serious	Serious [c]	Serious [e]	None	87	78	–	MD 1.50 lower (2.28 lower to 0.73 lower)	⊕◯◯◯ VERY LOW
**Weekly attack frequency**										
**3 Randomized**	Serious [f]	Serious [g]	Serious [c]	Very serious [h]	None	88	87	–	MD 2.66 lower (14.17 lower to 8.84 higher)	⊕◯◯◯ VERY LOW
**Recurrence rate**										
**2 Randomized (not pooled)**	Serious [i]	Not applicable	Serious [c]	Serious [j]	None	19/93 (20.4%)	38/82 (46.3%)	Zheng 2010: RR 0.555 (0.31–0.99); Si 2018: RR 0.30 (0.09–0.99)	–	⊕◯◯◯ VERY LOW
**Adverse events**										
**6 Randomized**	Serious [k]	Not serious	Not serious	Not serious	Publication bias strongly suspected [l]	26/264 (9.8%)	85/265 (32.1%)	RR 0.31 (0.21–0.46)	221 fewer per 1000 (from 173 fewer to 253 fewer)	⊕◯◯◯ VERY LOW

[a] Pooled effect statistically significant only in the high risk-of-bias subgroup (k = 16, P < 0.001); the some-concerns subgroup did not reach significance (k = 3, P = 0.12). Over 85% of VAS outcomes lacked

a specified recall period.

[b] I^2 = 93.1%; 95% prediction interval (−3.09 to 0.02) crossed zero. Meta-regression identified no significant moderator; residual heterogeneity exceeded 90% in all models.

[c] 22 of 23 trials used active pharmacological comparators without sham controls; acupuncture effects versus sham are consistently smaller than versus active controls. All trials conducted in China.

[d] Only one of three studies (Li 2024) specified a recall period. One study rated low risk, two rated some concerns.

[e] k = 3; total n = 165. Pooled estimate lost statistical significance upon removal of Zhang 2019 (P = 0.12) or Li 2024 (P = 0.08).

[f] Two of three contributing trials rated high risk of bias. No study used prospective pain diaries.

[g] I^2 = 78.3% with only three studies; sources of heterogeneity could not be investigated.

[h] 95% CI (−14.17 to 8.84) spans both substantial benefit and substantial harm; P = 0.424.

[i] Both trials rated some concerns. Neither used sham controls; recurrence ascertainment may be influenced by knowledge of group allocation.

[j] Each estimate derives from a single study at a different follow-up time point (6 months and 12 months). Both confidence intervals barely exclude 1.0.

[k] All contributing trials were open-label. Detection bias likely favours acupuncture for subjective adverse event reporting.

[l] 17 of 23 trials did not report adverse event data. Selective non-reporting of safety outcomes cannot be excluded.

Figure S1. Leave-one-out analysis for the effect on VAS scores

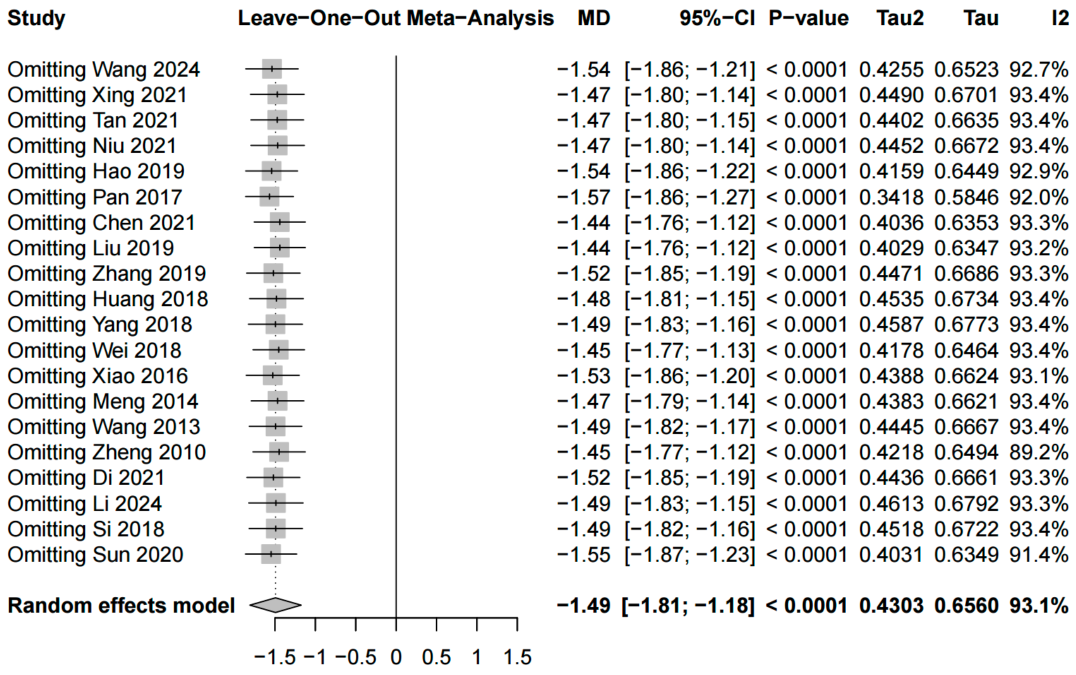

Figure S2. Funnel plot assessing publication bias for VAS pain intensity outcomes

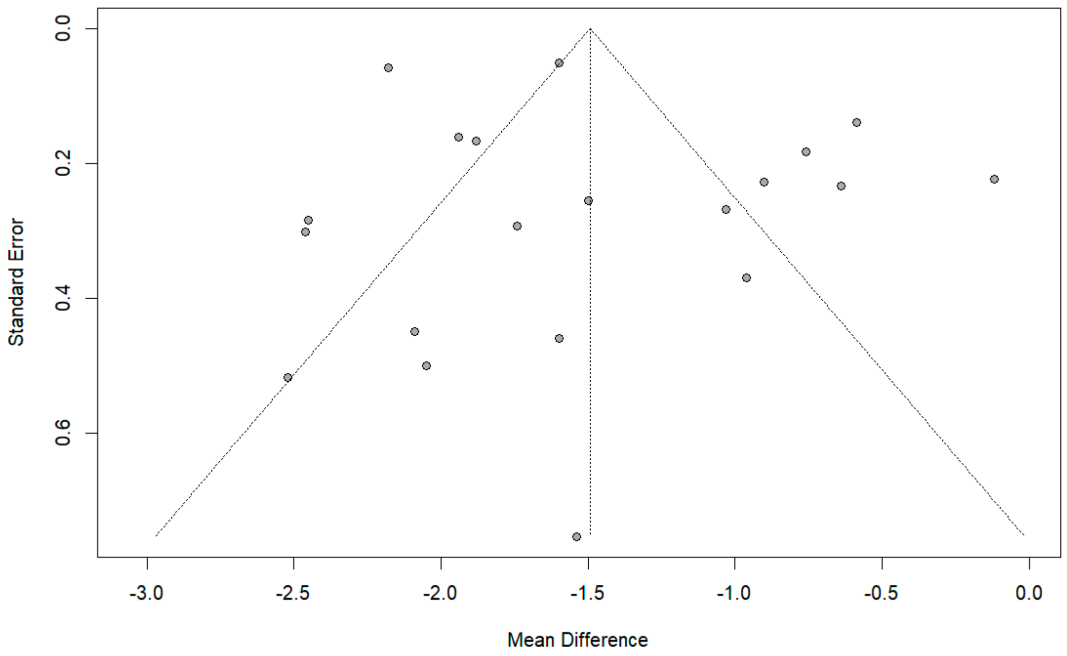

Figure S3. Forest plot of VAS pain intensity excluding the single sham-controlled trial

Study	Experimental Total	Experimental Mean	Experimental SD	Control Total	Control Mean	Control SD	MD	95%-CI	Weight
Wang 2024	30	-2.87	0.6938	30	-2.11	0.7255	-0.76	[-1.12; -0.40]	6.0%
Xing 2021	40	-5.53	0.8136	40	-3.65	0.6718	-1.88	[-2.21; -1.55]	6.0%
Tan 2021	33	-4.19	1.9209	33	-2.14	2.1348	-2.05	[-3.03; -1.07]	4.2%
Niu 2021	36	-5.55	0.3820	36	-3.61	0.8861	-1.94	[-2.26; -1.62]	6.1%
Hao 2019	27	-2.87	0.8329	27	-2.23	0.8850	-0.64	[-1.10; -0.18]	5.7%
Pan 2017	31	-2.83	0.8566	30	-2.71	0.8850	-0.12	[-0.56; 0.32]	5.8%
Chen 2021	40	-5.03	1.3874	40	-2.57	1.3077	-2.46	[-3.05; -1.87]	5.3%
Liu 2019	44	-4.97	1.3826	44	-2.52	1.2825	-2.45	[-3.01; -1.89]	5.4%
Zhang 2019	33	-4.00	1.0651	29	-2.97	1.0480	-1.03	[-1.56; -0.50]	5.5%
Huang 2018	32	-4.49	0.9968	31	-2.75	1.3093	-1.74	[-2.32; -1.16]	5.4%
Yang 2018	50	-3.10	1.2124	50	-1.60	1.3454	-1.50	[-2.00; -1.00]	5.6%
Wei 2018	31	-4.15	1.9018	31	-1.63	2.1680	-2.52	[-3.54; -1.50]	4.0%
Xiao 2016	50	-5.22	1.1715	50	-4.32	1.1152	-0.90	[-1.35; -0.45]	5.8%
Meng 2014	50	-4.11	1.9446	50	-2.02	2.5237	-2.09	[-2.97; -1.21]	4.4%
Wang 2013	19	-3.77	1.9689	20	-2.23	2.7042	-1.54	[-3.02; -0.06]	2.9%
Zheng 2010	60	-5.19	0.3500	60	-3.01	0.2900	-2.18	[-2.30; -2.06]	6.4%
Di 2021	30	-4.20	1.3187	30	-3.24	1.5416	-0.96	[-1.69; -0.23]	4.9%
Si 2018	33	-6.90	1.9733	33	-5.30	1.7608	-1.60	[-2.50; -0.70]	4.4%
Sun 2020	24	-1.12	0.3846	20	-0.53	0.5180	-0.59	[-0.86; -0.31]	6.1%
**Random-effects model (p = <1e-04)**	**693**			**684**			**-1.49**	**[-1.84; -1.14]**	**100.0%**
**Prediction interval**								**[-2.96; -0.02]**	

Heterogeneity: $I^2 = 93.3\%$, $\tau^2 = 0.4613$, $p < 0.0001$

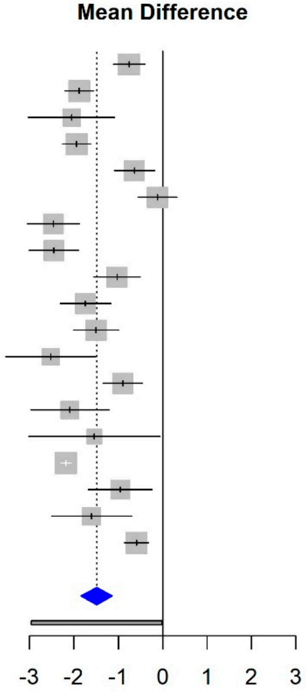

Figure S4. Leave-one-out analysis for the effect on VAS scores at 3-month follow-up

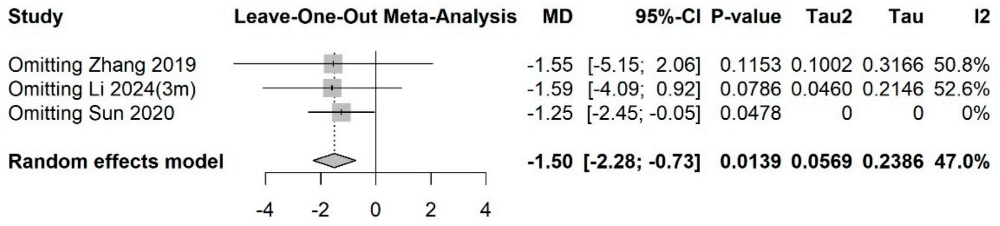

Figure S5. Forest plot of weekly attack frequency

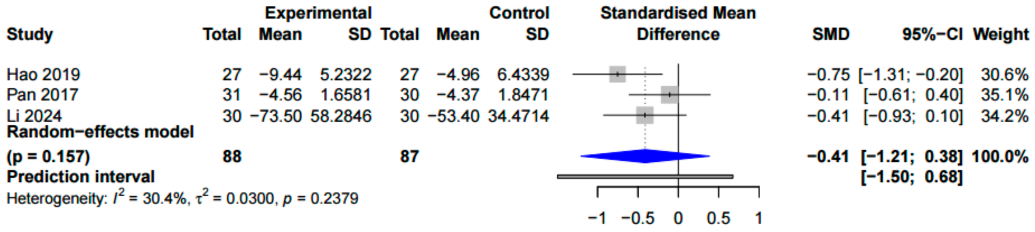

Figure S6. Leave-one-out analysis of weekly attack frequency

Study	Leave-One-Out Meta-Analysis	MD	95%-CI	P-value	Tau2	Tau	I2
Omitting Hao 2019		-6.31	[-123.05; 110.42]	0.6168	121.6800	11.0309	61.4%
Omitting Pan 2017		-7.48	[-85.65; 70.69]	0.4382	44.2958	6.6555	36.3%
Omitting Li 2024		-2.06	[-29.09; 24.97]	0.5100	7.8273	2.7977	85.1%
**Random effects model**		**-2.67**	**[-14.18; 8.84]**	**0.4236**	**9.3313**	**3.0547**	**78.3%**

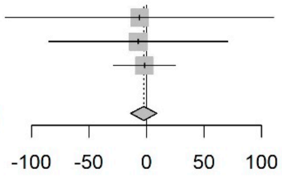

Supplement: Supplementary file 1 [file healthcare-14-01926-s001.zip › healthcare-4354498-supplementary.pdf]
